# [Corrigendum] Metastasis suppressor 1 expression in human ovarian cancer: The impact on cellular migration and metastasis

**DOI:** 10.3892/ijo.2025.5770

**Published:** 2025-07-02

**Authors:** Rong Liu, Tracey A. Martin, Nicola J. Jordan, Fiona Ruge, Lin Ye, Wen G. Jiang

Int J Oncol 47: 1429-1439, 2015; DOI: 10.3892/ijo.2015.3121

Subsequently to the publication of the above article, an interested reader drew to the authors' attention that, concerning the cell invasion assays shown in Fig. 5A on p. 1436, the 'WT' and 'pEF6' data panels contained apparently overlapping sections of data, such that these experiments were apparently derived from the same original source where the results of differently performed experiments were intended to have been portrayed.

After re-examining their original data, the authors have realized that the data panel in Fig. 5A for the 'pEF6' experiment was inadvertently selected incorrectly. The revised version of Fig. 5, showing all the correct data for Fig. 5A, is shown on the next page. The authors are grateful to the Editor of *International Journal of Oncology* for allowing them this opportunity to publish a Corrigendum, and all the authors agree with its publication. Furthermore, the authors apologize to the readership for any inconvenience caused.

## Figures and Tables

**Figure 5:**
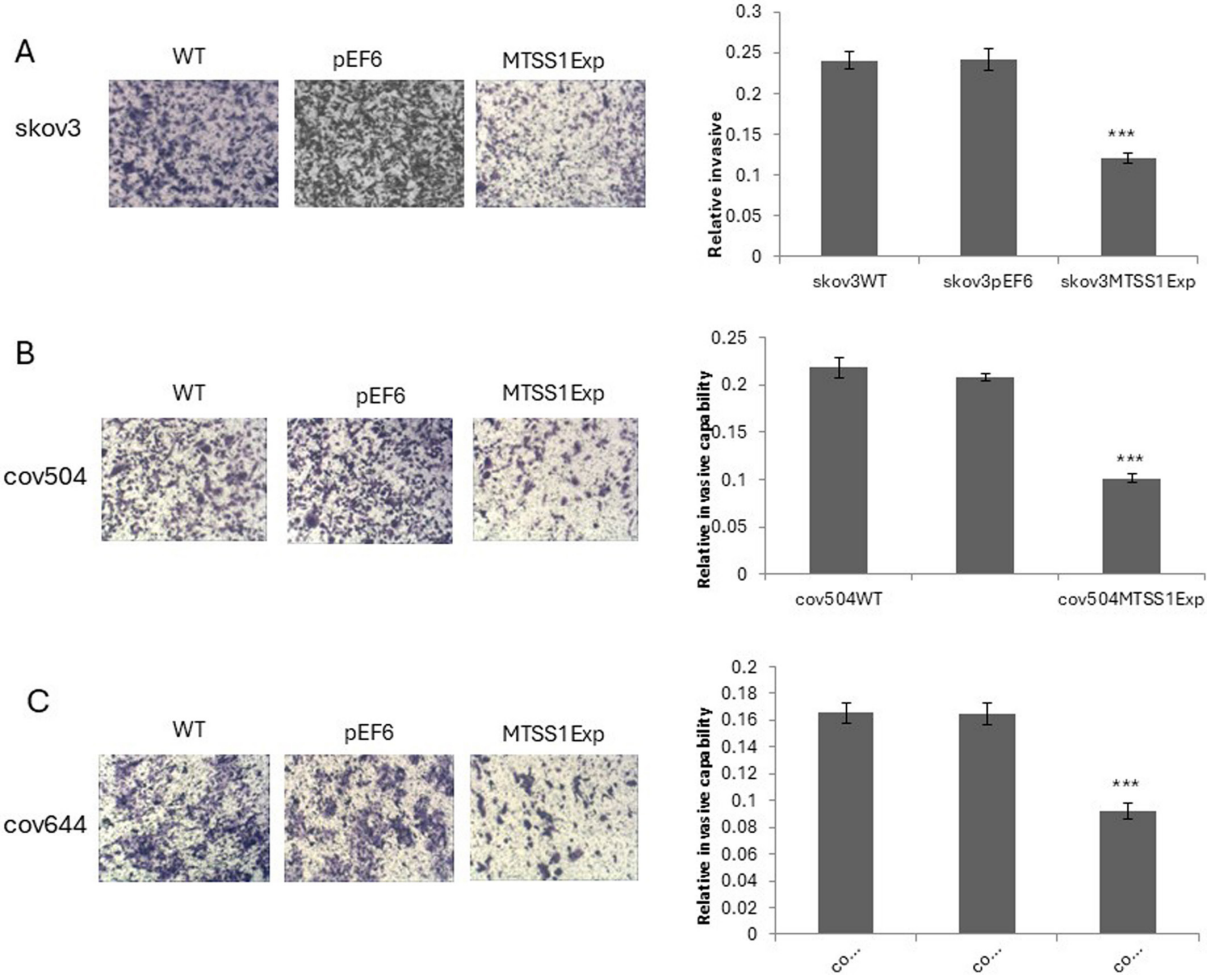
Effect of MTSS1 overexpression on the invasion of EOC cells. Cell invasion through Matrigel coated barrier was quantitated after fixing and staining invaded cells. Invasion capability was reduced in (A) SKOV3MTSS1Exp, (B) COV504MTSS1Exp and (C) COV644MTSS1Exp cells. Four fields of view/insert were photographed and counted. Images shown are from a typical field of view (×20 magnification). Data shown are mean ± SD cell number/membrane from 3 independent experiments. T-test compared MTSS1Exp vs. the controls for each cell line. ^***^P<0.001.

